# HIV Treatment-as-Prevention Research at a Crossroads

**DOI:** 10.1371/journal.pmed.1001654

**Published:** 2014-06-03

**Authors:** Till Bärnighausen, Nir Eyal, Daniel Wikler

**Affiliations:** 1 Harvard School of Public Health, Department of Global Health and Population, Boston, Massachusetts, United States of America; 2 Africa Centre for Health and Population Studies, University of KwaZulu-Natal, Mtubatuba, South Africa; 3 Harvard Medical School, Department of Global Health and Social Medicine, Boston, Massachusetts, United States of America

## Abstract

In light of changing WHO guidelines for HIV treatment, Till BÃ¤rnighausen and colleagues consider how large-scale HIV treatment-as-prevention trials can be adapted so that they can remain viable.

*Please see later in the article for the Editors' Summary*

**Summary Points**

- Randomized controlled trials of HIV treatment-as-prevention (TasP) are necessary to establish TasP effectiveness in general populations in sub-Saharan Africa.
- WHO's new HIV treatment guidelines inadvertently threaten the ongoing TasP trials in sub-Saharan Africa because they recommend substantially expanded HIV treatment eligibility.
- Historically, countries in the region have adopted WHO HIV treatment guidelines as national policies within two years of guideline publication. This time pattern is also emerging in the case of the new WHO guidelines: several sub-Saharan African countries are currently in the process of adopting the guidelines. If the countries hosting the TasP trials adopted the new WHO guidelines within the coming years, the trials in their original designs would become ethically impermissible, because they offer HIV treatment in the control arms under the more restrictive eligibility rules that are the current standard of care in the region. But offering the WHO-recommended expanded treatment standards in the control arms would likely render the trials underpowered.
- Fortunately, there are ways to generate rigorous evidence on TasP even if the new WHO guidelines are adopted. They include pooling results across trials and securing the agreement of governments to scale up expanded ART eligibility to communities in random order.

## New Guidelines

In June 2013, the WHO issued new guidelines for antiretroviral treatment (ART). The guidelines substantially expand eligibility for ART, recommending initiation at CD4 cell counts ≤500 cells/µl instead of at ≤350 cells/µl. For HIV-positive patients with active tuberculosis (TB) or hepatitis B, HIV-infected partners in serodiscordant couples, pregnant and breastfeeding women, and children younger than five years of age, ART is to begin immediately upon HIV diagnosis and irrespective of CD4 cell count or clinical stage [1].

While there has been vigorous debate regarding the strength of the evidence underlying some of the recommendations included in the WHO guidelines [2], as in the past, these guidelines are likely to be influential. Sub-Saharan African countries usually adopt WHO recommendations as national policies within a few years, while others anticipate them. For example, the WHO recommended ART initiation at CD4 cell count ≤350 cells/µl in 2010, when Ghana, Sierra Leone, Lesotho, Rwanda, Djibouti, Niger, and Tanzania had already adopted this standard; Guinea, Kenya, Malawi, South Africa, Swaziland, Zimbabwe, and Botswana followed within two years. Even before WHO's latest guideline change, Malawi and Zambia committed to providing lifelong ART to all pregnant and breastfeeding women; Rwanda's national guidelines recommend ART for all HIV-infected partners in serodiscordant relationships. Zimbabwe has declared that it will start initiating HIV-infected patients on ART when their CD4 cell counts drop below 500 cells/µl [3]; Zambia, Namibia and Swaziland are in the process of adopting the new ART initiation threshold for the general population; and South Africa is deliberating when to follow suit.

Given sufficient resources, adoption of the new WHO guidelines will likely lead to further reductions in the burden of HIV in countries severely affected by the epidemic. But there is also an unintended negative side-effect. The large-scale HIV Treatment-as-Prevention (TasP) trials planned or underway in sub-Saharan Africa are now at risk. The knowledge that the trials would generate would likely be critical to ensuring long-term government and donor enthusiasm for devoting extensive resources to HIV treatment. The case for such resource commitments would be much stronger if it were proven that HIV treatment can indeed substantially reduce HIV incidence in general populations in sub-Saharan Africa. We discuss policy options that would allow adoption of the new WHO guidelines while ensuring that we do not lose the historic opportunity to learn whether TasP works where it matters most.

## The TasP Trials

In addition to male medical circumcision, prevention of mother-to-child transmission (PMTCT) and pre-exposure prophylaxis (PrEP) [4], recent optimism for an “AIDS-free generation” has rested in large parts on the promise of TasP [5]. In TasP, ART is provided to all HIV-infected individuals upon HIV diagnosis, irrespective of CD4 cell count or clinical stage. The hope is that the suppression of viral loads in nearly everybody who is infected will prevent most onward transmissions of HIV. It has been shown that TasP nearly eliminates HIV transmissions in one particular population: HIV-uninfected partners in stable HIV-serodiscordant couples who have disclosed their HIV status to each other and are willing to jointly participate in an individually randomized controlled clinical trial [6]. However, TasP's potential for curbing the HIV epidemic in general populations with many different relationship types and different levels of care delivery and support remains an untested hypothesis, notwithstanding strong evidence on the preventive effect of ART under current guidelines from a population-based cohort study in rural South Africa [7] as well as results from several mathematical models predicting large TasP effects on HIV incidence [8],[ 9],[10].

To test the hypothesis that TasP can substantially reduce HIV incidence in general populations in sub-Saharan Africa, several multi-year cluster-randomized controlled trials are currently on-going or getting under way in South Africa, Zambia, and Botswana [11]–[13]. These trials have been funded with many tens of millions of dollars by the US National Institutes of Health (NIH), the Centers for Disease Control (CDC), the Bill & Melinda Gates Foundation, the French Agence National de Recherches sur le Sida et les Hépatites Viral (ANRS), and others. The trials are designed to compare the effect of TasP on HIV incidence against the practice of starting ART only in people with CD4 cell count ≤350 cells/µl or advanced clinical stages.

## How Adoption of WHO Guidelines Would Jeopardize the TasP Trials

If host countries adopt the new WHO threshold of CD4 cell count ≤500 cells/µl, current ethical standards will require that care given to patients enrolled as controls in the TasP trials be based on this threshold as well. For two decades, controversy has surrounded the standard of care offered to patients enrolled as controls in clinical trials in low-income countries. Opinions range from, at one extreme, the World Medical Association's Declaration of Helsinki, according to which new interventions “…must be tested against those of the best current proven intervention…” (§32) to those who would require only that care for controls meet the standard prevailing at the test site [14]. None of the parties to the dispute, however, has defended provision of care *inferior* to the care available locally. That is what was offered to trial subjects in the infamous Tuskegee Syphilis study and is one basis for its odious reputation.

The level of care for participants in the control arms of the TasP trials cannot be inferior to the care available locally. But the TasP trials were designed and powered assuming a CD4 cell count ≤350 cells/µl threshold. If sub-Saharan countries soon adopt the CD4 cell count ≤500 cells/µl threshold, the difference between the care provided in the TasP trials' intervention and control arms will probably be too small to detect statistically significant effects on HIV incidence. According to recent data, expanding general ART eligibility to patients at CD4 cell count ≤500/µl would roughly halve the number of people who are treatment-eligible in the TasP trials' intervention arms but ineligible in the control arms [15]. Moreover, the new WHO guidelines make all HIV-infected partners in serodiscordant relationships treatment-eligible regardless of CD4 cell count. About three-quarters of adults in sub-Saharan Africa report being in stable cohabiting relationships [16]–[18], and up to half of HIV-infected Africans in stable relationships have an HIV-uninfected partner [19]. Adoption of the new WHO guidelines thus would likely reduce by at least two-fifths the number of people who are treatment-eligible in the TasP trials' intervention arms but not in their control arms. Where the new guidelines are implemented, the difference in HIV incidence between the TasP trials' intervention and control arms will likely be reduced to a fraction of the difference under which the trials were originally powered. In this situation, data safety and monitoring boards (DSMBs) or policy makers may stop the trials; and even if they don't, the trials will likely fail in their primary aim to establish the effectiveness of TasP in general populations in sub-Saharan Africa because of insufficient power. With adoption of the new WHO guidelines as local standard now looming in the countries in which TasP trials take place, the premature or inconclusive end of the trials in their present form is thus predictable, raising the difficult question whether and how the trials should continue. Similar complications may arise for trials testing other ART-based HIV prevention strategies, such as interventions to prevent mother-to-child transmission. Here we focus on the case of TasP trials in the general population.

## Do We Still Need TasP Trials?

Policy makers, trialists, and members of the HIV community may wonder: Do we still need trials, now that ART initiation immediately after HIV diagnosis is considered so effective and safe for the individual patient's health that, since 2012, it is the United States standard of care [20]? The purpose of the trials, however, is not to establish the effectiveness or safety of early ART for already infected individuals, but to determine whether comprehensive TasP will reduce HIV incidence at the population level. A positive trial result would justify large increases in ART investment and TasP implementation in the countries worst-affected by the HIV epidemic.

If the incidence of HIV were to plummet wherever the new WHO guidelines are adopted, few would lament that TasP had not been validated in randomized control trials. However, if incidence continued to be high in some places after guideline adoption, effective HIV prevention strategies would remain necessary and yet we would not know whether TasP qualifies for this purpose. Absent trial data, TasP strategies might be wrongly discredited, with potential long-term damage to our ability to raise funds for the routine implementation of TasP, because governments and donors may not be willing to invest in expensive intervention strategies if their effectiveness has not been proven.

## Defensible TasP Trials?

How can rigorous scientific evidence on TasP effectiveness be obtained even as sub-Saharan countries are adopting the new guidelines? We present four alternatives. The first two are clearly unsatisfactory, in our view. The last two are tentative proposals that might deserve consideration. Even if none is satisfactory, they may prompt other proposals and serve as a starting point for an important debate.

1. *Compromising population health for science:* The trials could continue on their present course without asking controls to accept care below the local standard if policy makers in host nations deferred adoption of the new WHO guideline until the trials have been completed. However, assuming that the new WHO standard has substantial therapeutic value, the population health impact of such a move would make it clearly unethical.
2. *Expanding the trials:* The problem of underpowered trials could, in theory, be overcome either by greatly expanding the number of trial sites and participants or by lengthening the period of observation substantially beyond the originally planned duration. But it is unlikely that either of these options is feasible. At many tens of millions of dollars, the cost of the trials is already immense, and funding has been secured only with great effort. To ensure that the trials are not underpowered even as control arms are offered ART starting at CD4 cell counts ≤500/µl and treatment differences between intervention and control arms shrink, the scale and, accordingly, the budgets would likely have to be doubled or tripled. This is unlikely to happen.
3. *Pooling results:* Considered separately, the three trials that are currently under way in South Africa, Zambia, and Botswana will likely become underpowered as the new WHO guidelines are adopted and individuals in the control arms are offered ART under expanded eligibility. Jointly, however, the trials might remain sufficiently powered to detect significant incidence effects of TasP. Because of differences in trial design, it is currently unclear whether the results of the trials can be pooled. It is also uncertain whether the potential power gains from pooling will be sufficient to fully compensate for the expected power reductions following adoption of the new WHO guidelines. These questions should be urgently answered.
4. *Turning the trials into cluster-randomized scale-ups:* Even when governments commit to implementing the ≤500 CD4 cell count threshold, communities will know from experience that a national decision to adopt a new and higher standard of care is but the first step. In many communities, it may take many years before universal or near-universal access to care at the new standard is achieved. Indeed, even after a decade of vigorous ART scale-up in sub-Saharan Africa, WHO estimates that about 40% of currently eligible patients in the sub-continent are still not receiving ART [21]. In this proposal, the schedule for the gradual scale-up of the new WHO guidelines (or even of TasP) would be randomly assigned, creating the opportunity for a stepped-wedge randomized controlled study. The overall pace of the scale-up could remain unaffected. A key to scientific validity, randomization may also be ethically preferable to standard political decision-making on who receives the new standards first and who later. Randomization is valued for its impartiality [22]–[24], and it gives patients in remote rural areas, where rollout is more expensive and often comes last, an equal chance for ART [24],[25]. Such coordination between trialists and health policy-makers is a relatively new concept, but there have been some successful precedents [26].

## Conclusions

HIV TasP is one of our best current hopes for bringing the era of HIV to a close. Strong causal evidence that TasP works in general populations in sub-Saharan Africa, where the HIV epidemic is at its most severe, is still outstanding. This evidence, however, will be critical for ensuring that countries and donors will continue to provide the resources that are necessary to deliver near-universal ART coverage over the coming decades. Three large TasP trials that would generate this evidence are currently underway in sub-Saharan Africa. The adoption of the new WHO treatment guidelines, which recommend substantially expanded ART eligibility, would render the trials in their original designs unethical. Discussion is needed in order to allow some format of TasP trials to take place without delaying the adoption of the new WHO guidelines. We have mentioned several policy alternatives—some clearly unsatisfactory from an ethical or practical viewpoint, others more promising—in the hope of starting that discussion. More broadly, the case of the TasP trials and the WHO treatment guidelines is an example of the often difficult interaction between health policy and the scientific enterprise. Governments and international organizations demand strong evidence for policy formulation, but are commonly also compelled to act while evidence remains incomplete. As in the case of TasP, careful study design and well-coordinated policy implementation may allow major policy initiatives to go ahead without conclusive evidence, while preserving our ability to generate the evidence and, in doing so, ensuring the long-term success of the policies.

## Abbreviations

ART: antiretroviral treatment
TasP: treatment-as-prevention
